# Climate change, flooding, and HIV transmission in Africa: Potential relationships and a call for action

**DOI:** 10.1002/puh2.192

**Published:** 2024-06-16

**Authors:** Emmanuel Abiodun Oyinloye, Isaac Olushola Ogunkola, Yusuff Adebayo Adebisi, Iwatutu Joyce Adewole, Don Eliseo Lucero‐Prisno

**Affiliations:** ^1^ Department of Microbiology Faculty of Science Federal University Oye Ekiti Nigeria; ^2^ Youth RISE International Dublin Ireland; ^3^ Students for Sensible Drug Policy International Vienna Austria; ^4^ Nuffield Department of Population Health University of Oxford Oxford UK; ^5^ Gatefield Impact Abuja Nigeria; ^6^ Department of Global Health and Development London School of Hygiene and Tropical Medicine London UK

**Keywords:** Africa, antiretroviral therapy, climate change, flooding, human immunodeficiency virus, key population

## Abstract

The increasing effects of climate change have intensified floods globally, especially in Africa, where millions of people live in poverty and are highly vulnerable to flooding. Climate change disproportionately affects the vulnerable, who are least equipped to handle its consequences, by exacerbating their situation. One such consequence is the potential for increased human immunodeficiency virus (HIV) transmission. Africa has been disproportionately affected by the HIV epidemic. It now faces the additional challenge of a changing climate and floods, which are capable of increasing HIV transmission in Africa through several pathways. They can force population displacement and migration, leading to the expansion of sexual networks among people living with HIV (PLWHIV). They may also create conditions conducive to the spread of other infections. Floods can cause food insecurity, which can result in various sexual behaviors that expose people to HIV infection. As global warming is linked to a decrease in African food production capacity, the effect of food insecurity on HIV may be prominent in countries where transactional sexual means is a major route of HIV transmission. Floods can also hinder the provision of HIV services, such as pre‐ and postexposure prophylaxis and antiretroviral therapy distribution, which may worsen the health outcomes of PLWHIV and promote HIV transmission, particularly in rural and remote communities. It is crucial to develop a climate‐resilient framework, including education, sustained access to HIV services, and promotion of social welfare for HIV prevention and treatment, to address the complex relationship between HIV, floods, and climate change.

## INTRODUCTION

Flooding is common worldwide and has increased in intensity as deforestation and climate change worsen. Globally, 170 million people in extreme poverty are exposed to high flood risks. Additionally, 1.81 billion people worldwide live in areas with at least a 1% chance of experiencing a major flooding event in a year, particularly in developing nations. Flood exposure intersects with extreme poverty in Africa, especially in sub‐Saharan Africa, which affects 74.7 million people [1]. Heavy flooding typically arises in Africa from excessive rainfall, dam water discharge, rising sea levels, and overflowing rivers. Climate change contributes to the mass exodus of people from rural areas to developed cities with insufficient infrastructure, including vulnerable housing, inadequate drainage, and limited early warning systems, which exacerbated the impact of floods in Africa [2]. In 2022, flooding caused economic damage worth US$6.68 billion in Nigeria, whereas it caused over 5000 deaths and displaced 30,000 people in Libya in September 2023 [3, 4]. Climate change and floods disproportionately affect vulnerable populations, including orphans, refugees, disabled individuals, those with weakened immune systems, children, and women, disrupting access to essential resources such as food, water, and healthcare [5, 6].

Human immunodeficiency virus (HIV), a global pandemic since the 1980s, has claimed approximately 40 million lives. Of the 38.4 million people living with HIV (PLWHIV) worldwide in 2021, 15% are unaware of their status, which contributes significantly to HIV transmission [7]. Multiple studies have examined the influence of climate change on flooding and how HIV prevalence is impacted by the effects of extreme weather events, such as drought [6, 8]. Limited information exists on the potential connection between climate change, flooding, and HIV, especially in Africa, where 25.6 million PLWHIV live. Thus, this commentary aims to explore HIV transmission in Africa due to the impact of climate change and flooding.

## THE LINK BETWEEN CLIMATE CHANGE, FLOODING, AND HIV

**FIGURE 1:**
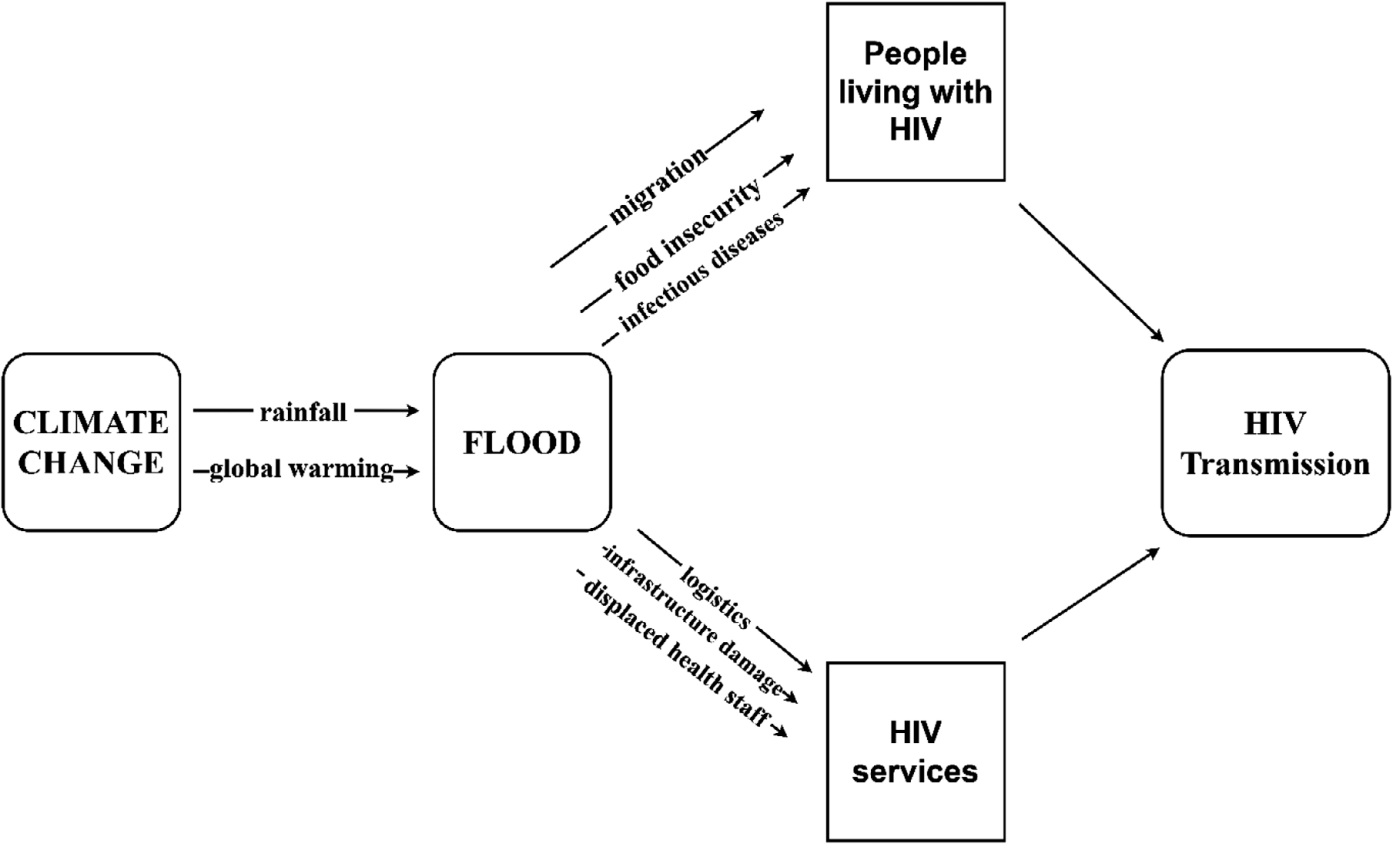
A framework of the complex relationship between climate change, flooding, and human immunodeficiency virus (HIV).

The complex connections between climate change, flooding, and HIV transmission, illustrated in Figure 1, provide further insight into the research findings of Nagata et al. and Lieber et al. [6, 8]. Climate change‐induced global warming is causing the expansion of seawater and melting of glaciers, resulting in rising sea levels and increased coastal flooding. Heavy rainfall, also linked to evaporation rates and precipitation patterns caused by global warming, exacerbates flooding worldwide.

Climate change and flooding are connected to HIV transmission through three major effects on PLWHIV: forced displacement and migration, food insecurity, and increased infections. Post‐flood family separation may exacerbate gender inequality, expose vulnerable children to sex abuse, and induce PLWHIV to notable HIV transmission risky behaviors such as survival sex, condom‐less sex, and intravenous drug use [5, 6, 8]. Due to climate change and floods, migration can result in the expansion of sexual networks and the transmission of HIV to new populations. Additionally, stigma and discrimination within refugee camps can hinder adherence to antiretroviral therapy (ART) medication schedules, disclosure of HIV status to new sexual partners, and usage of HIV services [5]. Social support is crucial to PLWHIV, as it helps them obtain information on HIV and acquired immunodeficiency syndrome (AIDS), advice, and assistance from family, religious, and community institutions, and alleviates their psychological anxiety. Loss of social support, especially for those in developing countries, can decrease the use of ART and HIV services [5]. Insecurity during flooding may drive increased physical, sexual, and gender‐based violence, thus contributing to HIV transmission risks.

Food insecurity may result from climate change, whereas floods may cause the destruction of farm products, the loss of livelihoods, and poverty. These may lead to transactional sex and extramarital affairs as coping mechanisms, leading to a rise in sexual partners. These patterns correspond with documented rises of 11% in sexually transmitted infections and a 12% increase in sexual partners following heavy rainfall and flood events [8]. Lack of food may also contribute to poor ART adherence and malnutrition, increasing the risk of vertical transmission of HIV.

Stress and poor water, sanitation, and hygiene at the refugee camps after flooding and other extreme weather events can lead to outbreaks of other infections, such as cholera and malaria. These infectious diseases stress the immune system of PLWHIV, heighten their viral loads, reverse the previous progress made by ART, and worsen the morbidity and mortality of PLWHIV [1, 8].

Floods can also increase HIV transmission through their effect on HIV services. Damages to infrastructure, reduced workforce, and increased workloads in the health sector during floods due to injuries and other flood‐related infections can reduce the quality and quantity of several HIV services [5, 8]. Overcrowded health facilities and disruption of road networks can toughen the procurement, distribution, and monitoring of ART, condoms, pre‐ and postexposure prophylaxis, and other essential services required for HIV transmission prevention and treatment [9].

## EFFECTS OF CLIMATE CHANGE AND FLOOD ON DRIVERS OF HIV IN AFRICA

Climate change and HIV disproportionately affect globally underdeveloped regions. Heavy rains, which tend to increase as the climate crisis continues, are a primary cause of floods across Africa and are linked to a 14% increase in the risk of HIV infection in sub‐Saharan Africa [8]. Thus, an increase in heavy rainfall in Africa may cause an increase in floods and a consequent increase in HIV transmission. Moreover, HIV services such as delivery of ART and exposure prophylaxis across Africa may be impeded during floods as the destruction of health facilities and roads will toughen the accessibility of Africans to HIV prevention services, especially in hard‐to‐reach communities and among key populations. African countries like Nigeria, with a high number of PLWHIV, as shown in Table 1, may see more HIV transmission as floods and climate change may promote sexual transmission among key populations, injecting drug use, vertical transmission, and other existing HIV transmission factors.

**TABLE 1:** Epidemiology of human immunodeficiency virus (HIV) and flood occurrence in selected African countries.

Country	Number of PLWHIV in 2022 [10]	Flooding event year	Impact of flood
Mozambique	2400,000	2013	Floods displaced 300,000, causing US$250 million in damage
		2015	Devastating floods resulted in 140 deaths, displaced 326,000, and caused US$371 million in damages
		2019	Cyclone Idai and flooding caused the death of about 600 people while affecting 1.85 million
		2022	Cyclone Gombe and floods caused 63 deaths and affected 736,000 people
		2023	Cyclone Freddy and intense rainfall caused heavy floods inundating 18,500 km^2^ and affecting 210,000 people
Nigeria	1900,000	2012	Severe floods affected 30 states, causing US$17 billion in economic loss and affecting 7.7 million people
		2018	Floods displaced and affected 210,000 and 1.9 million people, respectively
		2020	Floods affected 2353,000 people across different states
		2022	Heavy rainfall and dam water discharge caused floods, affecting 4.9 million people and US$9.12 billion in economic loss
Namibia	220,000	2009	Floods affected 677,000 people and caused 136.4 million physical damages
		2011	500,000 were affected by floods, displacing 60,000 and killing 65 people
Malawi	1000,000	2015	Floods affected 1.1 million individuals and resulted in 335 million damage and losses
		2019	It affected 975,000 people, causing US$220 million in damages
		2023	Cyclone Freddy and intense rainfall caused heavy floods affecting 2.2 million people and killing over 1200 individuals
Botswana	340,000	2013	Flooding displaced 400 and affected 4200 persons
Lesotho	270,000	2011	Floods displaced over 5000 people, killing 26 humans and 4700 animals
		2018	Heavy rains and hailstorms caused flash floods, with damage worth US$346,000, killing 12 people and displacing over 1400 others
South Africa	7600,000	2011	Heavy rainfall caused floods affecting 200,000 people, causing 40 deaths
		2022	Severe floods and landslides destroyed 12,000 houses, killing about 450 people and displacing over 40,000 others
Sierra Leone	77,000	2017	Floods and mudslides killed 300 people and left over 3000 homeless
		2022	The flood displaced about 13,000 people
Senegal	42,000	2012	11,400 homes were destroyed, whereas 287,000 people were affected by floods
		2016	Floods affected 12,000 people
		2020	Heavy rainfall caused floods, affecting 17,000 people

According to the World Bank, a 1.5–2°C increase in global temperature could lead to a 40%–80% reduction in African food production, creating an avenue for increased HIV transmission [6]. With 75 million Africans living in poverty already flood‐exposed, climate change through food insecurity and poverty may worsen the socioeconomic plight of several people across Africa, particularly vulnerable populations such as women living with HIV, thus creating more room for HIV transmission [1, 11]. In Malawi and Mozambique, where socioeconomic status is strongly linked to HIV transmission, HIV is most prevalent among women, and it is primarily transmitted through transactional sex, extramarital affairs, and high‐risk sexual behaviors, which may be exacerbated during floods [6, 8]. Mozambique and Malawi have 13% and 5.1% HIV prevalence, respectively. These countries frequently experience heavy floods caused by tropical cyclones and intense rainfall, leading to massive population displacement and economic damages, as shown in Table 1. In Malawi, the southern region experiences the highest population density and frequency of flooding events, coinciding with a higher HIV prevalence rate of 15% compared to 8% and 7% in the central and northern regions, respectively [12]. Similarly, gender discrimination and inequality may result from the pressure of climate change on limited natural resources, which may be a critical factor for Africa. For example, in Lesotho, reporting the second‐highest HIV prevalence globally, HIV transmission may be increased by climate change and flooding, worsening gender disparity, inequality, transgenerational sex, sex work, and other existing societal factors [8].

Furthermore, climate change exacerbates forced migration across Africa, thus promoting the spreading of HIV from immigrants to locals or vice versa. African countries where migration and risky sexual behaviors drive HIV transmission may see more HIV infections due to climate change. In Namibia, for example, multiple‐sex partnerships, intergenerational sex, and migration drive the HIV epidemic. With an HIV prevalence rate of 13.5% among adults, climate change and heavy floods can exacerbate these factors and worsen other existing socioeconomic challenges [8]. Floods in Namibia arise from heavy rainfall and poor infrastructure. Floods’ impact on public health and the economy, especially in areas with a high concentration of PLWHIV, may increase the risk of HIV transmission.

Lastly, Africa bears the highest burden of infectious diseases globally. Climate change may cause a spike in these diseases. Moreover, floods often allow for outbreaks of water and mosquito‐borne diseases; this may reduce people's natural immunity and increase HIV transmission risks.

## RECOMMENDATION AND CONCLUSION

Increased flooding is a key indicator of climate change and the most common natural disaster globally. Climate change and floods can worsen the welfare and health of people, especially PLWHIV, and they also lead to the loss of health services crucial to mitigating the spread of HIV. Natural disasters often disrupt healthcare systems and services, impede access to treatment, and exacerbate social inequalities—factors that can fuel the spread of HIV. Therefore, our strategies to combat HIV/AIDS must be integrated with our plans to address climate change, preserving and building on our progress in public health. To minimize the impact of floods on HIV transmission, PLWHIV should have continued access to ART, essential health services, food, and social support. Governments and organizations should spearhead the development of climate‐resilient, flood‐specific, and gender‐sensitive HIV prevention plans that incorporate telehealth services, comprehensive safe sex education, social welfare enhancement, and stigma‐reduction counseling. Further research is needed to identify other ways floods affect HIV transmission rates and develop new strategies to mitigate these effects. A metric to estimate the number of new HIV infections caused by floods should also be developed.

## AUTHOR CONTRIBUTIONS

*Conceptualization; methodology; writing—original draft*: Emmanuel Abiodun Oyinloye. *Writing—original draft*: Isaac Olushola Ogunkola and Iwatutu Joyce Adewole. *Writing—review and editing*: Yusuff Adebayo Adebisi and Don Eliseo Lucero‐Prisno.

## CONFLICT OF INTEREST STATEMENT

Isaac Olushola Ogunkola, Yusuff Adebayo Adebisi, and Don Eliseo Lucero‐Prisno III are Editorial Board members of Public Health Challenges and co‐authors of this article. They were excluded from editorial decision‐making related to the acceptance of this article for publication in the journal.

## FUNDING INFORMATION

The authors received no financial support for the research, authorship, and publication of this article.

## Data Availability

Data sharing does not apply to this article. All data analyzed in this study are publicly available and referenced within the manuscript. Supplementary data can be retrieved from relief web/UNOCHA reports via https://reliefweb.int/.
